# Short History of Malaria and Its Eradication in Italy With Short Notes on the Fight Against the Infection in the Mediterranean Basin

**DOI:** 10.4084/MJHID.2012.016

**Published:** 2012-03-10

**Authors:** Giancarlo Majori

**Affiliations:** Former Director Laboratory of Parasitology, Istituto Superiore di Sanità, Roma, Italy

## Abstract

In Italy at the end of 19^th^ Century, malaria cases amounted to 2 million with 15,000–20,000 deaths per year. Malignant tertian malaria was present in Central-Southern areas and in the islands. Early in the 20^th^ Century, the most important act of the Italian Parliament was the approval of laws regulating the production and free distribution of quinine and the promotion of measures aiming at the reduction of the larval breeding places of *Anopheline* vectors. The contribution from the Italian School of Malariology (Camillo Golgi, Ettore Marchiafava, Angelo Celli, Giovanni Battista Grassi, Amico Bignami, Giuseppe Bastianelli) to the discovery of the transmission’s mechanism of malaria was fundamental in fostering the initiatives of the Parliament of the Italian Kingdom. A program of cooperation for malaria control in Italy, supported by the Rockefeller Foundation started in 1924, with the establishment of the Experimental Station in Rome, transformed in 1934 into the National Institute of Public Health. Alberto Missiroli, Director of the Laboratory of Malariology, conducted laboratory and field research, that with the advent of DDT brought to Italy by the Allies at the end of the World War II, allowed him to plan a national campaign victorious against the secular scourge.

## Malaria in Italy and the Initial Control Interventions

**Figure 1:**
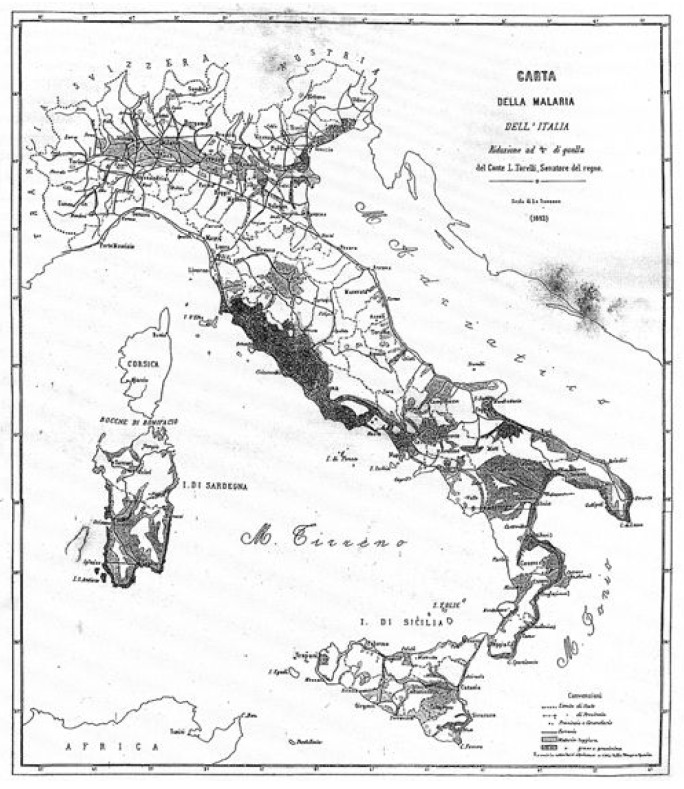
Map of malaria distribution in Italy. Torelli, Firenze, Pellas, 1882. (Reprinted from: *Il Laboratorio di Malariologia.* A cura di Giancarlo Majori e Federica Napolitani. Roma: Istituto Superiore di Sanità; 2010. (I beni storico-scientifici dell’Istituto Superiore di Sanità, Quaderno 5).

At the end of the 19^th^ Century, malaria in Italy was widespread in about one third of its territory where about 10% of the population lived. The total number of malaria deaths was in the range of 15,000–20,000 per year, and the malaria cases amounted to 2 million in a population of about 30 million. The situation in rural areas was often dramatic. In those years, the Health Service of the General Directorate of State Railways promoted a series of investigations on malaria morbidity to draw a map of the malaria endemic areas (Figure 1). In 1887, the year of the first compilation of health statistics in Italy, the malaria mortality rate was 710 per million inhabitants; it was higher in the Center (Maremma in Tuscany Region, Agro Romano), in the South and the islands as well, with a percentage of malignant tertian malaria that ranged between 20–30% of the cases. Malaria in Italy was severe along both the Tyrrhenian and Ionian coasts with the presence of *falciparum* malaria, in the low Veneto, Tuscany (Maremma), Southern provinces, and the Islands of Sardinia and Sicily. In the North there was the absolute presence of benign tertian and quartan malaria. About 2 million hectares of land were not cultivated because of malaria, especially in the areas mentioned above.

In Europe malaria was particularly present in countries of the Mediterranean basin and Eastern regions, including the European Russia. From the mid-nineteenth Century forward the process of regression of malaria started in North-Western European countries, as England and Holland, where the improvement of the health services in urban and rural areas progressed rapidly and resulted in a better control of the anopheline population. Throughout the first half of the 20^th^ Century, malaria in Europe underwent a continual reduction, thanks to a combination of improved economic situation and control measures regularly implemented.

In Italy, the reduction of malaria endemicity registered during the same period was mainly due to the approval of laws of great social importance. The most important act of the Italian Parliament was the approval of the law regulating the production and free distribution of quinine, followed by the promotion of measures aiming at the reduction of the larval breeding places of *Anopheline* vectors. Since 1898, especially because of the great political and social commitment of Angelo Celli, one of the fathers of the Italian Malariology - assisted by his wife Anne Fraentzel Celli (whose interesting diary was published under the pseudonym Heid in 1944)^1^ - the Italian Parliament discussed and adopted several regulations aimed to change the destiny of many regions affected by the disease.

**Figure 2:**
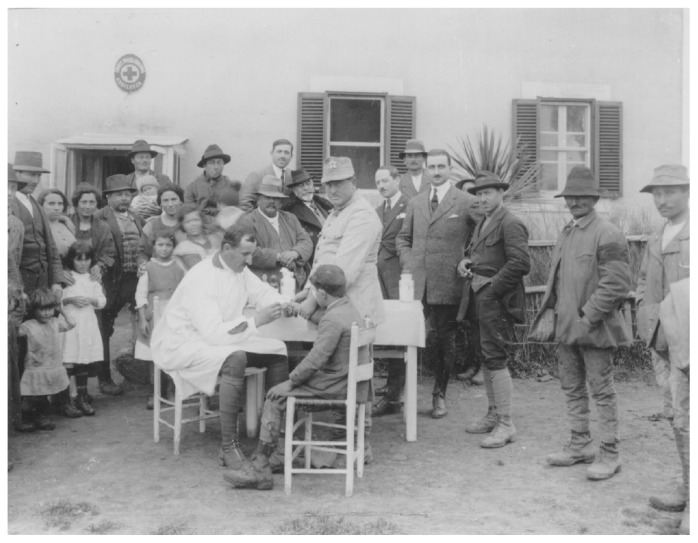
Blood collection for malaria diagnosis in rural population of Nettuno, Roma, 1920. (Reprinted from: *Il Laboratorio di Malariologia.* A cura di Giancarlo Majori e Federica Napolitani. Roma: Istituto Superiore di Sanità; 2010. (I beni storico-scientifici dell’Istituto Superiore di Sanità, Quaderno 5).

**Figure 3:**
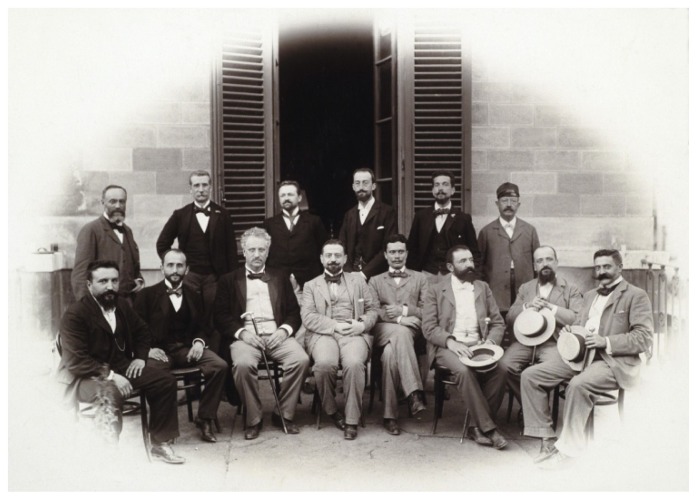
Roman School of Malariology at the end of 1800’s, malariologists and pathologists (sitting from the left: Amico Bignami and Giuseppe Bastianelli, first and fifth respectively; standing from the left: Ettore Marchiafava, Antonio Dionisi e Raffaele Bastianelli, second, third, and fifth, respectively). (Reprinted from: *Il Laboratorio di Malariologia.* A cura di Giancarlo Majori e Federica Napolitani. Roma: Istituto Superiore di Sanità; 2010. (I beni storico-scientifici dell’Istituto Superiore di Sanità, Quaderno 5).

In 1900, the law ensuring the national production and sale of the antimalarial quinine, the so called “Chinino di Stato”, state monopoly of quinine, was promulgated. In 1901, and later in 1904, the legislative measures regulating the free distribution of quinine to workers and settlers in malaria endemic areas to treat fever attacks were launched. The contribution of the local dispensaries and the capillary work of the district municipal doctors (Figure 2) were fundamental to the implementation of the legislative act. Furthermore, in 1923 the special law regulating the prophylactic use of quinine was approved, and it was harmonized with all services existing in the various Ministries relevant to public health matters. The contribution from the Italian School of Malariology (Camillo Golgi, Ettore Marchiafava, Angelo Celli, Giovanni Battista Grassi, Amico Bignami, Giuseppe Bastianelli) (Figure 3) to the discovery of the transmission’s mechanism of malaria was fundamental in fostering the initiatives of the Parliament of the Italian Kingdom.

Between September 1898 and February 1899, Grassi, concluded the researches on identification of malaria vectors (the mosquitoes belonging to the genus *Anopheles* are the *only incriminated* vectors), and together with Bignami and Bastianelli clarified the biological pattern of plasmodia from *Anopheles* mosquito to man in studies conducted in Santo Spirito Hospital in Rome using *Anopheles claviger* (synonym *Anopheles maculipennis*).^2^,^3^

Once the biological pattern of malaria transmission had been identified, all the possible breaking points in the transmission chain were considered to achieve the target of stopping it. But, a clear divergence of views between the two Schools of Malariology in Italy came out in selecting the existing malaria control methods: in an extreme fell those scientists who promoted the prophylactic and curative use of quinine, according to the method of radical treatment of malaria cases proposed by the famous bacteriologist Robert Koch (winner of the Nobel Prize in Medicine or Physiology in 1905) and, at the other extreme fell the malariologists who put major emphasis on vector control.

In the first quarter of the 1900’s in Italy the use of quinine was accompained by measures of larval control of vectors, always preferred to the control of adult mosquitoes. Initially, larviciding was carried out using petroleum derivatives, followed from 1921 by the “Paris Green” (a copper acetoarsenite) sprayed on the surface of the breeding places. Hydrocyanic acid sprayed against overwintering adult mosquitoes was also tested, but abandoned very soon because of the modest efficacy; the methods based on the use of pyrethrum extract and sulfur proved to be ineffective as well.

At the end of the World War I, the direct and indirect consequences of the conflict had led to a serious outbreak of the disease. To face the critical malaria situation, the control measures first implemented were land reclamation and larval control in channels, water puddles and ditches. In 1918, in order to train the Public Health Staff in charge of malaria control, a School of Rural Hygiene and Malaria Prophylaxis was established in Nettuno, a small town close to Rome. The School was under the authority of the Public Health Laboratories, national structures of the Public Health Directorate General of the Ministry of Interior of Italy.

## The Rockefeller Foundation in Italy

In 1913, John Davison Rockefeller, the founder of the Standard Oil Company, the American industrial economic empire, created in New York the charitable institution “Rockefeller Foundation”. The mission of the institution was to support the programs approved by the Society of Nations dealing with the improvement of health conditions of member countries. From 1915, the Foundation began to cope with malaria projects in the State of Mississippi and Arkansas, in collaboration with the United States Public Health Service and with local health authorities.

In 1921, after the discovery of the insecticidal action of the “Green of Paris” against the larvae of *Anopheles* mosquito^4^ an integrated approach coupling the control of malaria vectors and the prophylaxis with quinine was successfully tested, and in 1923 the League of Nations promoted an investigation in Europe on malaria endemicity and the use of quinine. On the basis of the malaria investigation carried out in Europe, the Foundation decided to support programs designed to identify and test the best method of malaria control.

In Italy, the initiative of the Rockefeller Foundation to support the fight against malaria began to substantiate on the occasion of the visit of Wickliffe Rose, Director of the International Health Board of the Rockefeller Foundation.^5^ According to Darwin H. Stapleton, former Director of the Rockefeller Archive Center, the cooperation with Italy was one of the most fruitful in the whole history of the Foundation.^6^ It should be stressed that the conditions requested to the country to obtain the Foundation’s grant were highly demanding, namely: (a) at the time of entry into force of the project, the activities of malaria control had to be already underway in the candidate country, and (b) the initiatives had to be of long duration, intended to be continued after the running out of the funding grant”.^7^

The program of cooperation between the Foundation and the Italian health authorities was designed to be an experimental malaria control model exportable to other European countries.^6^ Lewis Wendell Hackett, a public health doctor with previous experience in ancylostomiasis control in Central America, was appointed representative of the Rockefeller Foundation in Italy. Hackett arrived in Rome in January 1924, and immediately began to gather information on malaria control intervention in the country. In carrying out his enquire, Hackett met Alberto Missiroli, a research assistant of Bartolomeo Gosio, Director of the Italian Laboratories of Public Health. Since the first contact between Hackett and Missiroli a mutual esteem was born, prelude to the sincere friendship showed in the subsequent years. Missiroli, under Gosio’s supervision, developed a great interest in malaria control and followed the experiments on the efficacy of quinine conducted in Central Italy between 1919 and 1921. Hackett came to the conclusion that among all the Italian malariologists only Grassi and Missiroli were really interested in the scientific program he was undertaking. In fact, he wrote: *“…there are only two men in all Italy who are interesting themselves in the scientific study in malaria control, Drs Grassi and Missiroli who are working quite independently of each other, without program or budget, utilizing the spare hours which they can steal from their routine occupations…”*.^8^ Missiroli started to share the idea that the methods based on radical recovery of malaria infected patients were of little success. Hackett, according to his previous experience of malaria control matured in USA, considered vector control more promising, compared to the therapeutic approach. The common view in malaria control best strategy between Hackett and Missiroli flowed in a line of action seeking for the best methods of vector control.

## The Experimental Station for Malaria control in Italy

In August 1924, Hackett sent to the Rockefeller Foundation its project proposal of cooperation in Italy with these words: “*what Italy evidently needs, to be able to attack with any possibility of success its major public health problem, is undoubtedly a malaria bureau or division, under competent direction, with a separate budget, and a trained and whole-time scientific and subordinate personnel”*. Hackett strongly supported the connotation of independence of the Station “*in order to prevent it from becoming contaminated by the existing “network of intrigue” in Italian malariology*”.6

He conceived this new entity as a research center that would attract the international scientific community and play an important role in the training of malariologists. Hackett, working closely with Alberto Messea, Director General of Public Health, and with Missiroli, claimed that the future institute should deal initially with the collection of the epidemiological profiles of the various endemic areas in the country and with the definition of the habitat and behavior of the mosquito vector populations, in order to be able to assess the results of pilot malaria control trials. This attention to the specific areas stemmed from his idea that: *“malaria is a local and highly technical problem”*.^6^

Hackett received soon the agreement of the Foundation to his project proposal and quickly in 1924 his request of Liras 250,000 for the creation of the new structure was approved. The fund gradually rose to Liras 1,740,800 in 1927.^9^ The Governor of Rome put at the disposal of the cooperative project the palace “Farnesina” as headquarters. The activities started in 1925. The first peripheral Sections of the Station were established in Sardinia and Calabria regions, but later they were extended to other regions of Italy and the Sections amounted to a total of 16.^10^ The assignment of the Experimental Station was to provide Italy with adequate number of malariologists and technicians to tackle the problem of malaria control from a public health point of view, and to implement experimental protocols of malaria prevention and control in locations representative of particular epidemiological situations. The Rockefeller Foundation supported the Malaria Experimental Station in Italy from 1924 to 1934 to demonstrate that malaria control could be best achieved by anti-vector measures rather than by case treatment and prophylaxis with quinine.^6^

**Figure 4:**
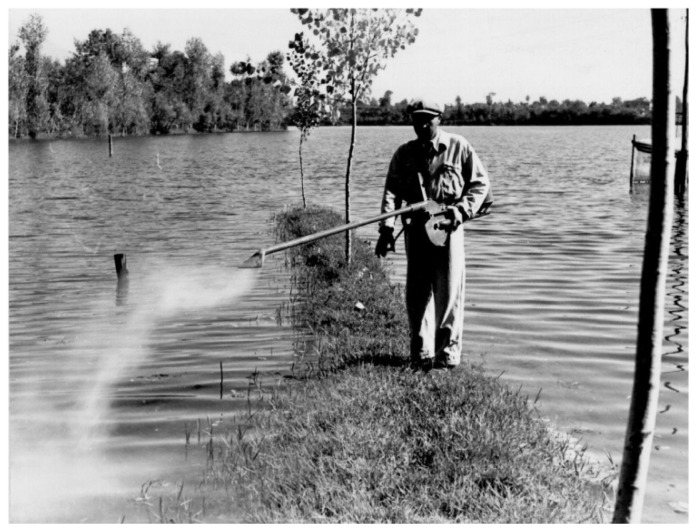
Spraying of “Paris Green”. Ceprano, Central Italy, 1939. (Reprinted from: *Il Laboratorio di Malariologia.* A cura di Giancarlo Majori e Federica Napolitani. Roma: Istituto Superiore di Sanità; 2010. (I beni storico-scientifici dell’Istituto Superiore di Sanità, Quaderno 5).

**Figure 5:**
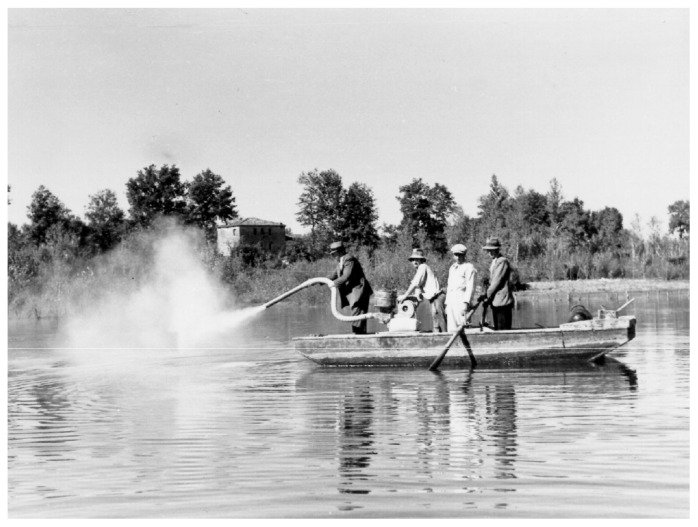
Spraying of “Paris Green” on boat. Ceprano, Central Italy, 1939. (Reprinted from: *Il Laboratorio di Malariologia.* A cura di Giancarlo Majori e Federica Napolitani. Roma: Istituto Superiore di Sanità; 2010. (I beni storico-scientifici dell’Istituto Superiore di Sanità, Quaderno 5).

The initial program was to assess whether simple anti-vectorial measures were sufficient to control malaria and if they could be adapted to the conditions and the economy existing in the European context.^11^ Missiroli, at that time Director of the School of Malariology of Nettuno, was appointed co-Director of the Experimental Station, together with Hackett as representative of the Foundation. Testing the efficacy of the antilarval compound “Green of Paris” was the first activity of the Experimental Station. The trials were carried out in different malarial areas of Central and Southern Italy (Figure 4 and Figure 5). The results showed a good efficacy, particularly in small larval breeding sites. The results of the preliminary experiments were presented by Hackett at the First International Congress on Malaria, held in Rome in October 1925, under the chairmanship of Ettore Marchiafava. The use of the “Green of Paris” was not considered the method of choice, and Missiroli stressed its inefficacy in vast rural areas with scattered houses. The malaria prophylaxis was adopted in Italy for a long time, but integrated with antilarval control methods.^12^ Hackett, in his volume “Malaria in Europe”,^13^ reports that the standard treatment, in use in 1925 in almost all malarial areas, consisted in 60 centigrams of quinine per day for eight weeks. But in 1926 at the beginning of the antimalarial campaign in Italy, the Experimental Station of Rome following the theory of Yorke and Macfie^14^ tested in Porto Torres, Sardinia Region, in parallel with antilarval treatment, a drug treatment of the local population with quinine: to half of the population was administered 1.0 gram of quinine per day for 6–7 days, while the other half followed the protocol of standard treatment, i.e. 0.6 grams a day for 6 weeks.^13^ The reduction in the length of treatment was a success in terms of compliance and clinical effects. Thus, all the Experimental Stations created by the Rockefeller Foundation in Southern Europe adopted, with minor variations, the “short-course treatment” also known as “Yorke’s treatment’. In 1933 the Commission of Experts of the Society of Nations recommended that treatment with quinine in any type of malaria should have been administered in not more than a week.^13^

Since 1926, the Experimental Station represented a reference center for international training and updating on advanced techniques in malaria research and control.

In the same years, in Italy integral reclamation works were implemented, on the basis of a legislation which pushed for (i) land reclamation; and (ii) health improvement aimed to enable farmers to reside permanently through the new assignment of farms.

The death of Grassi, which occurred in Rome on May 4, 1925, left a great vacuum of power, as Hackett impressively stressed in recalling his esteem to the scientist: *“He was a tremendous driving power in Italy in the field of malaria control and on his death there would be no one to carry on where if not for the program which we have undertaken to fulfill under the direction of Missiroli. Missiroli has the confidence and the friendship of everyone and I think the mantle could not have fallen on better shoulders”*.^7^

## The Institute of Malariology «Ettore Marchiafava»: origin and functions

The Institute “Ettore Marchiafava” was born as the transformation of the Superior School of Malariology, established in Rome in 1925 as an output of the aforementioned international Congress of malaria. The School was established with the aim of “*Promoting studies and training on all the medical problems related to malaria and on the reclamation and on the cultivation of marshy areas*”. His establishment was a consequence of the evolution of the methods of radical struggle against malaria by intervening, not only on the patient through quinine treatment, but at the same time on the territory with the definitive recovery of marshes with drainage of stagnant water. Initially, the School was directed by Professor Vittorio Ascoli and had its headquarters at the Medical Center of the Royal University of Rome. To Professor Ascoli succeded Professor Giuseppe Bastianelli who was appointed Director on March 25, 1931. With the new direction, the School was relocated from the Medical clinic to the IX pavilion of the Policlinico Umberto I. Six years on from its creation, the School was transformed into the “Institute of Malariology Ettore Marchiafava” because more suitable toward research. The Institute had the aim of teaching malariology and studying malaria. Prof. Giulio Raffaele was Director of the Instistute after Prof. Bastianelli, until the closure of the institute in 1967. In 1945, the High Commissioner of Health entrusted the Institute to carry out the antimalarial activities in the Province of Frosinone. The laboratory and field operations were implemented under the guidance of Prof. Alberto Coluzzi.^15^

## The enigma of anophelism without malaria

The enigma of “*anophelism without malaria*”, described by Roubaud,^16^ represented a great impasse in the understanding of malaria epidemiology: the partial or total lack of malaria in geographical areas where the anopheline vectors are very abundant contradicted the theoretical simplicity of the “Grassi’s law”, that’s*, infected man+anopheles mosquitoes = malaria*.^17^ This anomaly “ *anophelism without malaria*” struck Grassi himself. In fact, in 1919 the great malariologist had identified three typical malarious localities, all infested by anopheline mosquitoes but which were not affected by malaria in the same way. These localities were i) the gardens of Schito, near Naples, ii) Massarosa in Tuscany Maremma, and iii) Alberone, Lombardia. In 1921, Grassi, after repeated zoological and taxonomical observations in those areas, concluded his studies by assuming the existence of “races” of *Anopheles maculipennis* morphologically indistinguishable that do not bite humans, and that therefore cannot play a role of malaria vectors.

In 1926, Domenico Falleroni, a medical doctor, offered a step forward in solving the enigma by observing, without however speculating on the role of vector, that *Anopheles maculipennis*, morphologically indistinguishable in the adult stage, laid eggs with different shape and color of the esocorion.^18^ This observation opened the way to the identification of 6 varieties of *Anopheles maculipennis* complex. Missiroli and Hackett, in collaboration with the German entomologist Erich Martini, finally solved the enigma of the “anophelism without malaria” in 1933. They discovered that in Italian areas with low malaria endemicity, the dominant anopheline mosquitoes were those biting exclusively or preferably the cattle, and consequently not participating in the transmission of the disease. The observation was confirmed by comparing the intestinal contents of engorged mosquitoes, collected in animals shelters and in human dwellings, using immune sera according to the method developed in 1922 by American researchers in Louisiana.

*Anopheles maculipennis* complex consisted of species with different vectorial capacity, ranging from the very efficient *An. sacharovi* and *An. labranchiae*, through the poor vectors *An. atroparvus* and the *An. messeae*, to the definitely non-vector *Anopheles maculipennis* s.s.^19^ The biological explanation of the problem of anophelism without malaria made vector control appear again as a practical proposition and the means of overcoming the limits set by the presence of the inherently dangerous species of *Anopheles*.^20^

## The National Institute of Public Health

In 1928, on the occasion of the visit to the Experimental Station of Paul Frederick Russell, Director General of the International Health Board of the Rockefeller Foundation, Alessandro Messea, Director General of Public Health of the Ministry of the Interior, proposed to the Foundation to reorganize the Experimental Station for Malaria control and the National Laboratories of Public Health into an Institute of Hygiene. With similar objectives, Missiroli together with Hackett had already developed an operational plan for the transformation of the Experimental Station into a complex structure designed to be the National Laboratory of Public Health and a School of Public Health, similar to the Johns Hopkins University in Baltimore.^6^

In April 1930 the Rockefeller Foundation funded the construction of the Institute with a grant of $686,000 (equal to 12.5 million lire). The building was practically completed in December 1932.^7^ The Institute was inaugurated by Mussolini on the 21^st^ April 1934. The Institute included seven Laboratories (bacteriology, biology, malariology, chemistry, physics, sanitary engineering, epidemiology and prevention), a library and a museum. Prof. Missiroli was appointed Director of the Laboratory for the study of malaria, which subsequently changed the name into the Laboratory of Malariology. The Experimental Station with all its equipment was absorbed by the Laboratory. In 1941 the Institute of Public Health became the Istituto Superiore di Sanità (ISS) (Royal Decree October 17 1941-XIX, n. 1265), and expanded the activities of clinical, laboratory and field research in malaria and other public health subjects.

Under the direction of Missiroli, the primary task of the Laboratory of Malariology was efficacy tests of new chemical products for malaria prevention and control. From 1935, new antimalarial products (acridinin derivatives) were tested on animals and were carried out extensive research on plasmodia (*Plasmodium elongatum*) and on their development, together with studies on the genetics of *An. maculipennis* complex. The use of atebrin and plasmochin, in treatment and prophylaxis of malaria, was tested in Sardinia by Ezio Mosna.^21^

The commitment of the Laboratory also included field tests of antilarval products, monitoring the regional experimental stations and monitoring the work of land reclamation. Mosna, which will be appointed Director of the Laboratory after Missiroli, introduced studies on malariotherapy, after a period of training in 1934 at the Neuropsychiatric Clinic of the University of Vienna.^22^

## The malaria situation in Italy before World War II

Toward the end of ‘30s, the malaria situation in Italy was considerably improved. In 1939, malaria cases in the Pontine Marshes fell almost to zero. In Veneto the malariological indices in most affected provinces of Venice and Rovigo had decreased, and the cases of *P. perniciosus* malaria were dramatically reduced.^23^ The same happened, although to a lesser extent, in the areas of the Central and Southern Italy and the islands, where the consumption of quinine and other subsidiary drugs decreased with the decrease in the number of cases. *The World War II.* From June 10, 1940, with the entry of Italy into the world conflict, the malaria prophylaxis began to suffer from financial difficulties due to the economic restrictions. Therefore, the control of the *Anopheles* populations suffered a setback for the shortage of fuel that was rationed all over Italy. Other difficulties underwent when the “Green of Paris” was proposed, due to the unavailability of copper. In this context, in order to prevent the resurgence of malaria among the populations in the Pontine Marshes and the Agro Romano and in other areas of Italy, Missiroli and his collaborators did everything possible to keep the healthcare organization.^24^,^15^ The malaria situation became even more serious compared to what occurred during and after the first world war. At that time the troops were operating in the Alps, while now they were in Central and Southern regions, historically much more malaria endemic areas. The war caused the destruction of every public service so that the operations of prophylaxis were forcedly interrupted. Missiroli took the direction of the malaria control activities in the areas of Ostia and Fiumicino and Maccarese (July 1943); he was also appointed commissioner of the Antimalaria Committee of Latina.

In October 1943 the German Army, in order to prevent or slow down the advance of the Allied Powers (the landings of Allies at Anzio, just south of Rome, took place on 22 January 1944) began to flood the areas of the Agro Pontino and Agro Romano, laboriously reclaimed. In a letter to Prof. G. Marotta, Director General of the Istituto Superiore di Sanità, dated November 29, 1943, Missiroli gave news of the flooding of Ostia, Fiumicino and Maccarese, as well as the Agro Pontino and expressed concern for the high risk of resumption of malaria transmission in the summer season of 1944.^25^ In the same letter, Missiroli communicated to the Director General, that on November 14, 1943 the German government had sent to Rome Prof. Erich Martini, of the University of Hamburg and Prof. F. Rodenwaldt, of the University of Heidelberg, “*to study the possible damages resulting from the flooding the reclaimed lands”* and informed the Director General about his recommendations addressed to the German colleagues, namely i) to avoid the contamination of soils with salt water, ii) not to damage the dewatering pumps, iii) not confiscate the dairy cattle and iv) not divert the health personnel from its functions. Martini and Rodenwaldt promised to Missiroli that they “*would have recommended the German Command to take in serious account the recommendations presented”*. Martini, before leaving, assured Missiroli that he would have used his scientific influence to avoid flooding with brackish water and also he would have asked the Germans to respect the Geneva Convention for the civil and military health-care personnel. Finally, Martini and Missiroli agreed each other that the Laboratory of Malariology would have maintained contacts with the German military health command.^26^

Unfortunately, in spite of all the expectations, what happened was that after the initial flooding the retreating German Army did blast 50,000 mines in Latina Province, causing the destruction of the draining pumps and razing to the ground the civil buildings. The ditch system of the Pontine marshes had been completely disrupted. This tactic was used everywhere by the retreating Germans. The aerial and naval bombardments opened tens of thousands of craters, which soon became as many larval breeding sites of anopheline mosquitoes. In addition to the creation of breeding places, the whole Province was deprived of the mechanical protection of homes from entering mosquitoes, as the explosions of grenades and bombs had torn all windows mosquito nets. All the old larval breeding sites in wetland and along river shores became covered by abundant vegetation developed during two years of complete stop of maintenance works.^27^ The report written by Missiroli on August 24, 1944, and sent to the Secretary of State and the General Directorate of Public Health, presented the general malaria situation and the commitment of the Laboratory. During the conflict, the Laboratory of Malariology continued his work of research, despite imaginable difficulties of the moment. The activity, in fact, took place mainly in the laboratory and to a very limited extent in the field. In 1943 and 1944 continued to organize the training and refresher courses on malaria for the national and military personnel.^28^

## The Antimalaria Campaign in the Post-War Period

### First attempts of malaria control

At the end of World War II, malaria reinvaded vast areas of Italy, mainly the Central and Southern regions, major islands and North-eastern coastal areas, with offshoots of hypoendemicity in the Pianura Padana. The three vectors were *Anopheles labranchiae* Falleroni and *An. sacharovi* Favre, both belonging to the so-called *maculipennis* complex, and *An. superpictus* Grassi. *An. labranchiae* was the principal vector in the central and southern coastal areas, Sicily, and Sardinia. In the two islands, the species was found as high as 1,000 meters above sea level. *An. sacharovi* was present along much of the coastal area and in Sardinia, but was most important as vector in the plains of the northeastern Adriatic coast, where *An. labranchiae* was absent. *An. superpictus* played a role of secondary vector in central and southern Italy and Sicily. In some interior areas of the Pianura Padana, where none of the three vectors was present, low levels of endemicity were probably maintained by other species belonging to the *maculipennis* complex.

The malariologists of the Istituto Superiore di Sanità, on the basis of the poor financial and technical resources available, managed to reorganize the control operations in Italy liberated. In Northern Italy, the “Istituto Interprovinciale antimalarico delle Venezie” had kept its autonomy and performed malaria control operations, in line with other Regional antimalaria committees, i.e. Sicily and Sardinia. Initially, “Paris Green”, where available, was used, together with a rigorous prophylaxis with mepacrine, especially in the Pontine Marshes, where a severe malaria epidemic caused in 1945 a mortality of 1‰ and a morbidity 72%.

## The Arrival of the Allies to Italy, the Advent of DDT, and the Five-Year Malaria Eradication Program

Dichlorodiphenyltrichloroethane (DDT) was first added to U.S. Army supply lists in May 1943, and was used for the first time against a petechial thyphus epidemics in Naples in 1944. Studies in USA at the Orlando, Florida, Laboratory of the Bureau of Entomology and Plant Quarantine demonstrated beyond question that this new insecticide had tremendous possibilities not only against lice but also against several other noxious insects, such as mosquitoes and houseflies.^29^ With the help of the War Production Board, the new insecticide was put immediately into large production. The first field test, in which residual DDT was applied to the interior walls of all houses and outbuildings against *Anopheles* vectors began in Italy in the spring of 1944. This experiment was carried out in the town of Castel Volturno, north of Naples, by the Malaria Control Demonstration Unit of the Malaria Control Branch of the Public Health Sub-Commission, Allied Control Commission, Italy. Spraying began on 17 May 1944, and this experiment, together with a second one started later in the Tiber Delta area, lasted 2 years.^30^ The Malaria Control Demonstration Unit, under the direction of Dr. F. L. Soper, consisted largely of members of the Rockefeller Foundation Health Commission who had brilliantly carried out their first assignment, that of assisting in typhus control in Naples.^31^

In 1945 in the delta of the River Tiber (Ostia and Isola Sacra), the Health Commission of the Rockefeller Foundation, at the request of medical authorities of the US army, had undertaken, with the contribution of the ISS, an experiment to test the efficacy of DDT applied on the internal walls of buildings and animal shelters against *Anopheles labranchiae*. Missiroli, exploiting his personal relations, managed to get by the allied Command 70 quintals of DDT technical product and the quantity of oil needed to obtain a solution of 5% DDT to use in laboratory and field tests. Missiroli planned to spray DDT at the dosage of 1–2 gram/square meter, and after months the sprayed surfaces still retained the insecticide against the indigenous *Anopheles* mosquitoes. The results were announced by Missiroli in a famous conference held on November 25, 1944, just a few months after the Italy’s liberation, in the Chamber of Commerce and Agriculture in Rome. He impressed the audience by announcing a new ambitious objectives that would have brought the eradication of malaria from Italy *“.....our aspiration is no longer limited to reduce the number of new infections and to treat malaria: today we tend to liberate Italy from this disease, aware that the scientific means available will allow to us to reach the target in a relatively short time*”.

**Figure 6:**
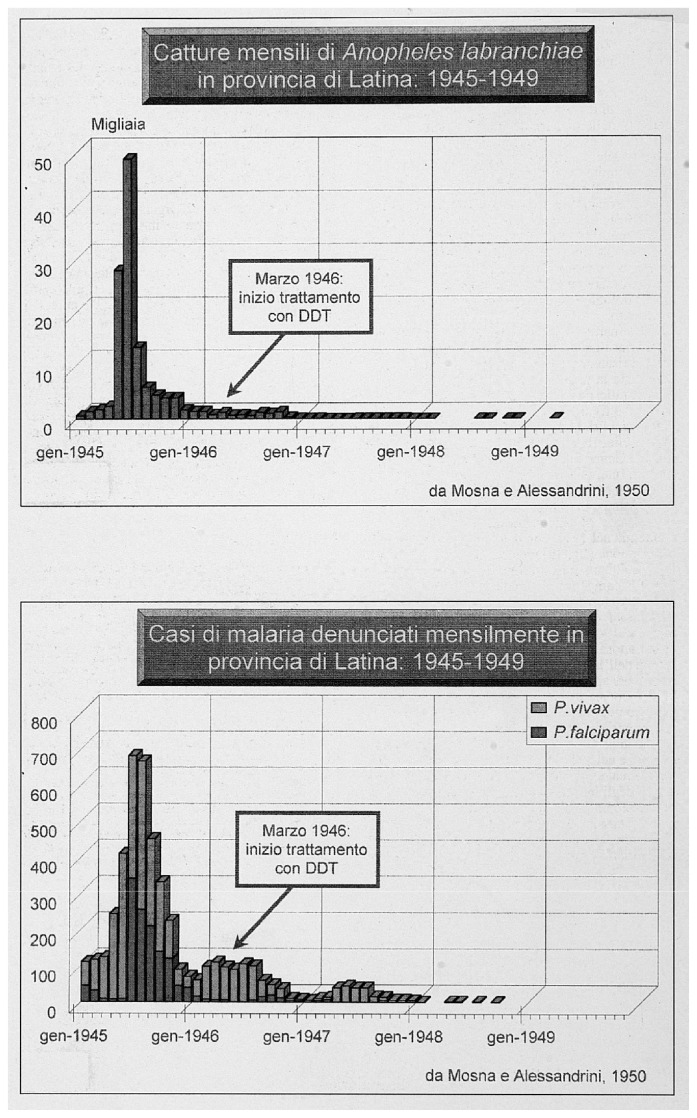
Results of DDT application. Monthly collection of *Anopheles labranchiae* and malaria cases in Latina Province from 1945 to 1949. Reprinted from: *Il Laboratorio di Malariologia.* A cura di Giancarlo Majori e Federica Napolitani. Roma: Istituto Superiore di Sanità; 2010. (I beni storico-scientifici dell’Istituto Superiore di Sanità, Quaderno 5).

**Figure 7:**
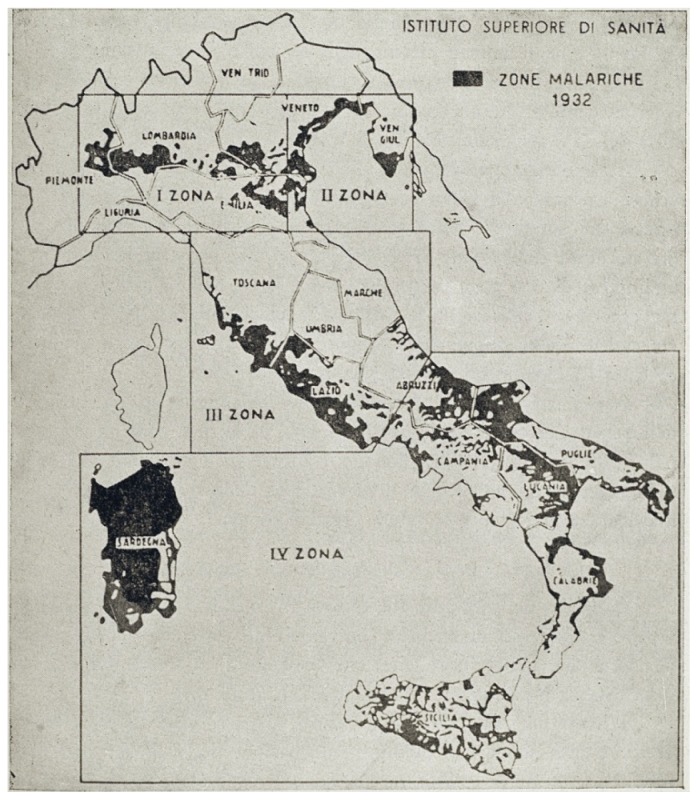
The Five-year Malaria Eradication Program in Italy, 1947–1951. The four epidemiological zones. Reprinted from: *Il Laboratorio di Malariologia.* A cura di Giancarlo Majori e Federica Napolitani. Roma: Istituto Superiore di Sanità; 2010. (I beni storico-scientifici dell’Istituto Superiore di Sanità, Quaderno 5).

**Figure 8:**
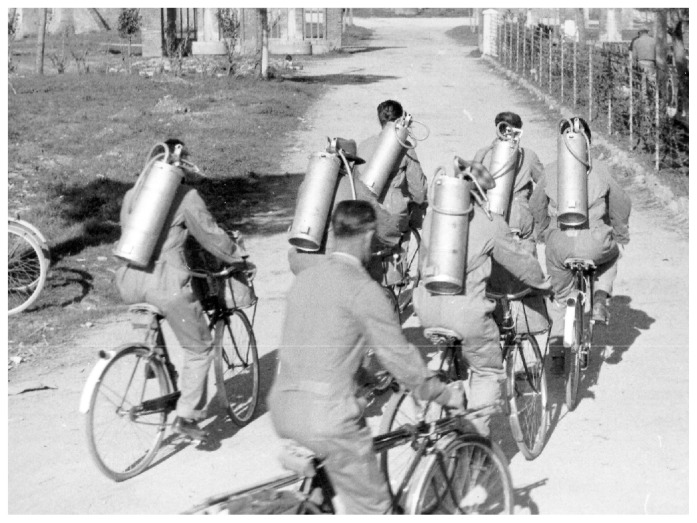
A DDT spraying team on bycicles. 1947. Reprinted from: *Il Laboratorio di Malariologia.* A cura di Giancarlo Majori e Federica Napolitani. Roma: Istituto Superiore di Sanità; 2010. (I beni storico-scientifici dell’Istituto Superiore di Sanità, Quaderno 5).

Missiroli, helped by the United Nations Relief and Rehabilitation Administration (UNRRA) decided to realize for the first time an experiment to control malaria by exploiting the long residual insecticidal activity of DDT against adult mosquitoes, applying it on rural homes and animal shelters. Ezio Mosna was responsible for organizing a large-scale experiment that beginning the June 5, 1945 in the plain of Fondi in the Province of Latina. The first positive results arrived in July 1945: the data on collection of *Anopheles* mosquitoes in the highly endemic area showed the mosquito population nearly disappeared (Figure 6).^27^ In January 1946, Missiroli announced a five-year plan of action designed to eradicate malaria from Italy. The plan divided Italy into four areas, following the biological characteristics of the anopheline population present in each of them (Figure 7). The campaign for the eradication of malaria from the whole national territory began in 1947 and ended virtually in 1948, with the total interruption of transmission of *falciparum* malaria. Indoor treatment with DDT (2 g of active ingredient per m^2^) of houses, stables, shelters, and all other rural structures continued into the mid-1950s and even later in some hyperendemic areas. (Figure 8). The results of the first two years of the year of DDT campaign were communicated by Missiroli in the IV Congress of Tropical Medicine and Malaria in Washington in 1948 under his vice-presidency.^32^ On that occasion Missiroli received unconditional awards. The continuation of the vector control campaign led to a further lowering of anopheline density and kept the interruption of the transmission of malaria.^33^ The last endemic focus of *P. vivax* was reported in the province of Palermo, Sicily, in 1956, followed by sporadic cases in the same province in 1962. The World Health Organization declared Italy free from malaria on November 17, 1970. Since then, almost all reported cases have been imported.

## The Sardinian Project on Malaria Vector Eradication

In 1947 the Rockefeller Foundation started a project aiming at the eradication of malaria vectors from Sardinia. The Sardinian Project was an “all-out attempt to eradicate *Anopheles* mosquitoes from the island…(*omissis*)…to determine whether or not the species eradication technique is applicable to the problem of malaria control in the Mediterranean region”.^34^ The project was managed by the “Ente Regionale per la Lotta Anti-anofelica in Sardegna” (ERLAAS) with the financial contribution and under the technical direction of the Foundation. In contrast to what was done in the continental territory of Italy, DDT was used not only inside buildings but also outside on all the waters where could larval stages growth. The result was that on the close of operation in 1950, malaria transmission had been eliminated, but *An. labranchiae* still existed. The Sardinian project was a failure: the result was just the interruption of malaria transmission with a considerably higher spending than in other parts of Italy and with a sensitive environmental pollution.

## Malaria in Southern Europe

In Europe malaria was present in countries of the Mediterranean basin and Eastern regions, including the European Russia. From the mid-nineteenth Century forward the process of regression of malaria started in North-Western in other parts of Italy and with a sensitive environmental pollution.

European countries, as England and Holland, where the improvement of the health services in urban and rural areas progressed rapidly and resulted in a better control of the *Anopheline* population. As mentioned before, in the first half of the 20^th^ Century, malaria in Europe underwent a constant reduction, thanks to a combination of improved economic situation and control measures regularly implemented. The success in Italy against the historical scourge was preceded and followed by other success stories occurred in countries of the Mediterranean Basin, each of them strictly obtained according to specific epidemiological conditions and local technical and political commitment.

In 1900 malaria patients could be found throughout Europe. Hackett in 1937 explained the “spontaneous” disappearance of malaria from some areas by stating that “*as a result of a more scientific agriculture the map of malaria in Europe is thus steadily contracting toward the limits set for it by the spread of the anophelines which we have defined as inherently dangerous. Beyond this there can be no further “natural regression”*. That limit was the distribution of the major vectors *An. labranchiae* and *An. sacharovi*, while malaria had disappeared spontaneously from the areas of distribution of *An. messeae* and had resisted, but eventually subsided, in some foci of *An. atroparvus*.^20^ In Northern Europe, towards the end of the Nineteenth century, malaria began to disappear because of environmental changes for agricultural practices and stabling of cattle with an attractive effect on the local vector, i.e. *An. atroparvus* in Denmarks.^35^,^36^ In early 1900s the “drug approach” with quinine and the “anti-vectorial measures” dominated the malaria control activities in Southern Europe.

In Spain, in 1900, Ian McDonald, a Scottish malariologist employed by a British mining company, carried out the first study to support the role of anopheline mosquito in malaria transmission;^37^ the arrival in Spain of Gustavo Pittaluga, an Italian pupil of Grassi, gave further impetus to the anophelic-parasitic explanation of malaria. Pittaluga, in 1906 got a position at the National Institute of Hygiene, directed by the Nobel Prize Santiago Ramon y Cajal. From 1911 held the chair of Parasitology and Tropical Pathology at the University of Madrid, by contributing to reformulate the concept of “malaria environment” as the sum of the conditions favorable to the spread of the disease. He planned anti-malaria measures in Spain based on prophylactic administration of quinine and mechanical protection of the population. In August 1920, a Committee for the Draining of Malaria Areas was set up, and under Pittaluga’s input, it became the Central Anti-Malaria Committee.^37^ Wars influenced the trend of malaria control. The Spanish Civil War in 1939 and later the Second World War produced a severe recrudescence of malaria all over in the country.^34^,^38^ The introduction of DDT and the use of antimalarials produced an immediate drop off of malaria incidence and the final eradication from Spain in 1962.

Malaria was endemic in Portugal in several areas at the beginning of the 1900’s. In Portugal malaria control programs initiated in 1931 in the Tagus river basin, in areas with extensive rice cultivation. In 1933, a study financed by the Rockefeller Foundation, showed that malaria occurred in the alluvial valleys of the main rivers, but especially in rice field areas where *An. atroparvus* had its breeding places. The malaria situation induced the Rockefeller Foundation to establish a malaria research station, which became in 1938 an Institute for Malaria Research and Training (Instituto de Malariologia).^39^ Plans for eradication of the disease initiated in the 1950’s with DDT spraying, and by 1958 the transmission of the infection was interrupted in nearly all areas of Portugal. The country was placed in the maintenance phase of malaria eradication and the certification of malaria eradication was confirmed by the WHO in 1973.^40^

In Greece, malaria eradication campaign started in 1946 with intensive indoor spraying of DDT against the anopheline populations, in particular the principal malaria vector *Anopheles sacharovi*. In spite of the appearance of resistance of *An. sacharovi* to DDT at the initial application rounds, the vector population was satisfactorily controlled.^41^,^42^ The spraying operations were discontinued at the end of ‘60s. Resurgence of *An. sacharovi* was reported 12 years after malaria eradication.^43^

## The Global Malaria Eradication Program of WHO

In 1955 the WHO launched the Global Malaria Eradication program is a worldwide campaign for the eradication of malaria based on the use of DDT and other insecticides with residual activity applied inside the housing against vector *Anopheles* mosquitoes and on the use of antimalarial drugs for the elimination of plasmodia in humans. The campaign brought, toward the end of the 1960s, the eradication of malaria in all developed countries where was endemic (Mediterranean countries, many regions of the tropics, etc.) and produced the interruption of malaria transmission in most areas of the tropical Asia and Latin America (e.g. in Brazil the number of cases decreased from 6 million to 37,000). With regard to Africa, partial limited campaign took place only in three countries since, on the basis of the results of pilot projects, it was not considered feasible to extend the program to the rest of the continent.^44^ The initial results of the campaign were extraordinary, but in the following years there were no further replication of the improvements, and on the contrary, the insecticide resistance of the vectors to DDT and the plasmodia to chloroquine, influenced heavily the performance of the eradication program, to the point that many areas ex-endemic were reinvaded by malaria.

In 1969 the WHO abandoned the strategy of the eradication and replaced it with that of the control, i.e. a planned reduction of morbidity and mortality. In 1992 drew up a new strategy with emphasis on early diagnosis and immediate treatment in the context of programs managed by the basic health care system.^20^ Many countries such as Thailand, China, Brazil, Solomon Islands, Philippines, Vietnam, obtained good results in terms of control. For many others, and especially for those in sub-Saharan Africa, the malaria situation is still critical. There were an estimated 216 million episodes of malaria and 655,000 malaria deaths in 2010, of which 91% were in Sub-Saharan Africa. The estimated incidence of malaria globally has reduced by 17% since 2000 and malaria-specific mortality rate by 26%. These rates of decline are lower than internationally agreed targets for 2010, but nonetheless they represent a major achievement.^45^
